# Supplementary material for: Modeling glioblastoma heterogeneity as a dynamic network of cell states
Source: Mol Syst Biol. 2021 Sep 16;17(9):e10105. doi: 10.15252/msb.202010105 (PMC8444284; doi:10.15252/msb.202010105)
Supplement: Supplementary file 6 — Source Data for Figure 5 [file MSB-17-e10105-s004.zip › Figure5A_sourcedata/GSEA_3071/nosigdb_stateIV.GseaPreranked.1622795164626/GAROFANO_IAVARONE_NEU_SUBTYPE.html]

Details for gene set GAROFANO\_IAVARONE\_NEU\_SUBTYPE[GSEA]

|  || Dataset | state53071 |
| Phenotype | NoPhenotypeAvailable |
| Upregulated in class | na\_pos |
| GeneSet | GAROFANO\_IAVARONE\_NEU\_SUBTYPE |
| Enrichment Score (ES) | 0.5033306 |
| Normalized Enrichment Score (NES) | 2.9130542 |
| Nominal p-value | 0.0 |
| FDR q-value | 0.0 |
| FWER p-Value | 0.0 |
Table: GSEA Results Summary

  

Fig 1: Enrichment plot: GAROFANO\_IAVARONE\_NEU\_SUBTYPE      
 Profile of the Running ES Score & Positions of GeneSet Members on the Rank Ordered List

  

| PROBE | GENE SYMBOL | GENE\_TITLE | RANK IN GENE LIST | RANK METRIC SCORE | RUNNING ES | CORE ENRICHMENT || 1 | DCX |  |  | 4 | 2.752 | 0.0279 | Yes |
| 2 | CELF2 |  |  | 63 | 1.766 | 0.0350 | Yes |
| 3 | DCLK2 |  |  | 74 | 1.704 | 0.0508 | Yes |
| 4 | SLC35F1 |  |  | 78 | 1.647 | 0.0674 | Yes |
| 5 | PTPRO |  |  | 80 | 1.628 | 0.0842 | Yes |
| 6 | NRXN2 |  |  | 83 | 1.621 | 0.1007 | Yes |
| 7 | PCSK1N |  |  | 110 | 1.507 | 0.1113 | Yes |
| 8 | NCAM1 |  |  | 129 | 1.456 | 0.1230 | Yes |
| 9 | LSAMP |  |  | 130 | 1.454 | 0.1381 | Yes |
| 10 | CHD7 |  |  | 144 | 1.386 | 0.1500 | Yes |
| 11 | GRIA2 |  |  | 160 | 1.347 | 0.1612 | Yes |
| 12 | BCL11B |  |  | 163 | 1.343 | 0.1748 | Yes |
| 13 | ZEB2 |  |  | 164 | 1.339 | 0.1887 | Yes |
| 14 | TNIK |  |  | 172 | 1.312 | 0.2010 | Yes |
| 15 | NCAN |  |  | 181 | 1.298 | 0.2130 | Yes |
| 16 | SOX6 |  |  | 207 | 1.244 | 0.2211 | Yes |
| 17 | SHC3 |  |  | 215 | 1.218 | 0.2324 | Yes |
| 18 | SATB1 |  |  | 222 | 1.203 | 0.2438 | Yes |
| 19 | KIF5C |  |  | 240 | 1.170 | 0.2527 | Yes |
| 20 | FHOD3 |  |  | 245 | 1.161 | 0.2640 | Yes |
| 21 | PFN2 |  |  | 270 | 1.107 | 0.2709 | Yes |
| 22 | GPC2 |  |  | 292 | 1.053 | 0.2778 | Yes |
| 23 | PODXL2 |  |  | 326 | 0.992 | 0.2817 | Yes |
| 24 | FOXN3 |  |  | 348 | 0.977 | 0.2878 | Yes |
| 25 | CACNG4 |  |  | 371 | 0.941 | 0.2933 | Yes |
| 26 | RUFY3 |  |  | 376 | 0.929 | 0.3022 | Yes |
| 27 | SOX4 |  |  | 377 | 0.928 | 0.3119 | Yes |
| 28 | ATCAY |  |  | 397 | 0.910 | 0.3177 | Yes |
| 29 | MARCKSL1 |  |  | 417 | 0.888 | 0.3232 | Yes |
| 30 | KLHL24 |  |  | 419 | 0.885 | 0.3322 | Yes |
| 31 | RAB6B |  |  | 424 | 0.877 | 0.3406 | Yes |
| 32 | CRMP1 |  |  | 438 | 0.866 | 0.3471 | Yes |
| 33 | SBK1 |  |  | 439 | 0.865 | 0.3561 | Yes |
| 34 | DAAM1 |  |  | 444 | 0.860 | 0.3643 | Yes |
| 35 | CLIP3 |  |  | 451 | 0.854 | 0.3720 | Yes |
| 36 | TCEAL2 |  |  | 464 | 0.845 | 0.3785 | Yes |
| 37 | DCC |  |  | 472 | 0.835 | 0.3858 | Yes |
| 38 | RPAIN |  |  | 493 | 0.822 | 0.3905 | Yes |
| 39 | ABAT |  |  | 515 | 0.805 | 0.3948 | Yes |
| 40 | ZMYM2 |  |  | 542 | 0.773 | 0.3978 | Yes |
| 41 | RERE |  |  | 568 | 0.750 | 0.4008 | Yes |
| 42 | WSB1 |  |  | 578 | 0.741 | 0.4067 | Yes |
| 43 | TMOD2 |  |  | 591 | 0.732 | 0.4120 | Yes |
| 44 | RNF165 |  |  | 606 | 0.725 | 0.4169 | Yes |
| 45 | ARHGEF9 |  |  | 688 | 0.667 | 0.4081 | Yes |
| 46 | ATF7IP |  |  | 711 | 0.653 | 0.4106 | Yes |
| 47 | POU2F1 |  |  | 718 | 0.649 | 0.4162 | Yes |
| 48 | ITM2C |  |  | 724 | 0.645 | 0.4219 | Yes |
| 49 | ZNF711 |  |  | 774 | 0.618 | 0.4189 | Yes |
| 50 | KIDINS220 |  |  | 777 | 0.616 | 0.4249 | Yes |
| 51 | SRGAP3 |  |  | 789 | 0.609 | 0.4291 | Yes |
| 52 | MAPT |  |  | 844 | 0.583 | 0.4247 | Yes |
| 53 | NKD1 |  |  | 847 | 0.581 | 0.4303 | Yes |
| 54 | NAV2 |  |  | 865 | 0.574 | 0.4330 | Yes |
| 55 | NIN |  |  | 869 | 0.574 | 0.4384 | Yes |
| 56 | STMN4 |  |  | 876 | 0.570 | 0.4432 | Yes |
| 57 | PAG1 |  |  | 882 | 0.568 | 0.4481 | Yes |
| 58 | SLITRK2 |  |  | 889 | 0.565 | 0.4529 | Yes |
| 59 | HIPK2 |  |  | 896 | 0.563 | 0.4575 | Yes |
| 60 | NKTR |  |  | 914 | 0.555 | 0.4600 | Yes |
| 61 | SLC6A1 |  |  | 927 | 0.549 | 0.4634 | Yes |
| 62 | PHLPP1 |  |  | 977 | 0.533 | 0.4595 | Yes |
| 63 | SENP7 |  |  | 989 | 0.526 | 0.4628 | Yes |
| 64 | FAM49A |  |  | 1016 | 0.516 | 0.4631 | Yes |
| 65 | CLASP2 |  |  | 1017 | 0.515 | 0.4685 | Yes |
| 66 | GNG2 |  |  | 1019 | 0.515 | 0.4736 | Yes |
| 67 | CCDC88C |  |  | 1032 | 0.511 | 0.4766 | Yes |
| 68 | EVL |  |  | 1047 | 0.506 | 0.4792 | Yes |
| 69 | ZNF462 |  |  | 1111 | 0.488 | 0.4720 | Yes |
| 70 | HMBOX1 |  |  | 1113 | 0.488 | 0.4769 | Yes |
| 71 | CPT1C |  |  | 1135 | 0.484 | 0.4779 | Yes |
| 72 | JPH4 |  |  | 1137 | 0.484 | 0.4827 | Yes |
| 73 | GADD45G |  |  | 1147 | 0.481 | 0.4860 | Yes |
| 74 | NAV1 |  |  | 1170 | 0.474 | 0.4867 | Yes |
| 75 | TSPAN7 |  |  | 1181 | 0.471 | 0.4896 | Yes |
| 76 | PLXNB1 |  |  | 1207 | 0.463 | 0.4896 | Yes |
| 77 | GSK3B |  |  | 1227 | 0.457 | 0.4907 | Yes |
| 78 | PHYHIPL |  |  | 1270 | 0.447 | 0.4872 | Yes |
| 79 | TMEM170B |  |  | 1281 | 0.444 | 0.4898 | Yes |
| 80 | ALDOC |  |  | 1284 | 0.443 | 0.4941 | Yes |
| 81 | DISC1 |  |  | 1293 | 0.441 | 0.4971 | Yes |
| 82 | KIF1A |  |  | 1304 | 0.440 | 0.4997 | Yes |
| 83 | TMEFF1 |  |  | 1310 | 0.438 | 0.5033 | Yes |
| 84 | BMP7 |  |  | 1368 | 0.423 | 0.4967 | No |
| 85 | AKT3 |  |  | 1417 | 0.412 | 0.4916 | No |
| 86 | RIMS3 |  |  | 1418 | 0.412 | 0.4959 | No |
| 87 | FAM110B |  |  | 1440 | 0.405 | 0.4961 | No |
| 88 | SPPL3 |  |  | 1462 | 0.399 | 0.4961 | No |
| 89 | MNX1 |  |  | 1478 | 0.396 | 0.4973 | No |
| 90 | NLGN2 |  |  | 1534 | 0.381 | 0.4906 | No |
| 91 | TMSB15A |  |  | 1540 | 0.381 | 0.4936 | No |
| 92 | GRIA4 |  |  | 1545 | 0.379 | 0.4968 | No |
| 93 | MYO16 |  |  | 1621 | 0.364 | 0.4860 | No |
| 94 | NCOA2 |  |  | 1640 | 0.360 | 0.4863 | No |
| 95 | REV3L |  |  | 1664 | 0.356 | 0.4855 | No |
| 96 | LMBR1L |  |  | 1687 | 0.352 | 0.4849 | No |
| 97 | GSTA4 |  |  | 1702 | 0.349 | 0.4858 | No |
| 98 | MTSS1 |  |  | 1708 | 0.348 | 0.4885 | No |
| 99 | ACVR2B |  |  | 1725 | 0.345 | 0.4890 | No |
| 100 | RAB33A |  |  | 1760 | 0.337 | 0.4859 | No |
| 101 | YLPM1 |  |  | 1824 | 0.327 | 0.4770 | No |
| 102 | CDO1 |  |  | 1827 | 0.327 | 0.4801 | No |
| 103 | FAM117B |  |  | 1853 | 0.323 | 0.4786 | No |
| 104 | DCLK1 |  |  | 1860 | 0.321 | 0.4807 | No |
| 105 | RAP2A |  |  | 1903 | 0.314 | 0.4758 | No |
| 106 | PELI1 |  |  | 1921 | 0.310 | 0.4758 | No |
| 107 | NXPH1 |  |  | 1950 | 0.306 | 0.4735 | No |
| 108 | MBTD1 |  |  | 1956 | 0.305 | 0.4757 | No |
| 109 | LRCH2 |  |  | 1962 | 0.304 | 0.4779 | No |
| 110 | ELMO1 |  |  | 1966 | 0.303 | 0.4805 | No |
| 111 | ABCC5 |  |  | 1983 | 0.300 | 0.4805 | No |
| 112 | SPAST |  |  | 1986 | 0.300 | 0.4832 | No |
| 113 | ZNF248 |  |  | 2009 | 0.295 | 0.4820 | No |
| 114 | CASK |  |  | 2014 | 0.294 | 0.4843 | No |
| 115 | KIAA0895L |  |  | 2040 | 0.290 | 0.4825 | No |
| 116 | CDK5R1 |  |  | 2126 | 0.280 | 0.4689 | No |
| 117 | CCDC28B |  |  | 2142 | 0.278 | 0.4689 | No |
| 118 | RTN3 |  |  | 2160 | 0.274 | 0.4684 | No |
| 119 | ENHO |  |  | 2173 | 0.272 | 0.4689 | No |
| 120 | FAM13C |  |  | 2235 | 0.264 | 0.4598 | No |
| 121 | XYLT1 |  |  | 2244 | 0.263 | 0.4610 | No |
| 122 | DUSP26 |  |  | 2260 | 0.260 | 0.4608 | No |
| 123 | KSR1 |  |  | 2262 | 0.260 | 0.4633 | No |
| 124 | SNAP25 |  |  | 2289 | 0.257 | 0.4609 | No |
| 125 | MYT1 |  |  | 2292 | 0.257 | 0.4632 | No |
| 126 | PLCH2 |  |  | 2411 | -0.259 | 0.4430 | No |
| 127 | ZMIZ1 |  |  | 2414 | -0.259 | 0.4453 | No |
| 128 | CLN8 |  |  | 2508 | -0.270 | 0.4301 | No |
| 129 | PTPRD |  |  | 2553 | -0.275 | 0.4244 | No |
| 130 | TSHZ1 |  |  | 2622 | -0.285 | 0.4141 | No |
| 131 | MED13L |  |  | 2690 | -0.294 | 0.4042 | No |
| 132 | PARP8 |  |  | 2903 | -0.319 | 0.3663 | No |
| 133 | MIIP |  |  | 3086 | -0.343 | 0.3345 | No |
| 134 | RNF144A |  |  | 3276 | -0.371 | 0.3016 | No |
| 135 | PCDH7 |  |  | 3576 | -0.423 | 0.2479 | No |
| 136 | PRKAB2 |  |  | 3773 | -0.460 | 0.2146 | No |
| 137 | SUSD4 |  |  | 4056 | -0.526 | 0.1653 | No |
| 138 | CSMD2 |  |  | 4098 | -0.540 | 0.1630 | No |
| 139 | MAPRE2 |  |  | 4160 | -0.557 | 0.1569 | No |
| 140 | RIMS1 |  |  | 4201 | -0.568 | 0.1551 | No |
| 141 | NEGR1 |  |  | 4509 | -0.685 | 0.1026 | No |
| 142 | EPHB2 |  |  | 4657 | -0.768 | 0.0820 | No |
| 143 | POU2F2 |  |  | 4811 | -0.876 | 0.0614 | No |
| 144 | KLRC2 |  |  | 5122 | -1.340 | 0.0151 | No |
| 145 | BASP1 |  |  | 5203 | -1.665 | 0.0169 | No |
Table: GSEA details [plain text format]

  

Fig 2: GAROFANO\_IAVARONE\_NEU\_SUBTYPE: Random ES distribution      
 Gene set null distribution of ES for **GAROFANO\_IAVARONE\_NEU\_SUBTYPE**
